# The Unsatisfactory Panel System

**Published:** 1913-10-04

**Authors:** 


### OUR NATIONAL INSURANCE ACT DEPARTMENT.

### LXIX.—The Unsatisfactory Panel System.

During the past few weeks a number of circumstances have combined to show the unsatisfactory nature of the present administration of medical benefit under the panel system. The system appears to be unsatisfactory to all parties concerned. The holiday season has led insured persons to complain that they have been unable to obtain treatment whilst temporarily absent from home. Approved societies have been more insistent than ever in complaining that the present administration of medical benefit leads to malingering, and doctors now seem to have good cause for complaint at the delay in settling their accounts on the capitation basis, and of the difficulty in determining whether an insured person is on their lists or not.

### Medical Benefit when Away from Home.

Endeavours have been made by the Insurance Commissioners in their instructions to Committees to modify the method of administration as far as possible so that the advantage of medical treatment and attendance shall be made available for insured persons while temporarily absent from home, and to meet the requirements of that comparatively large class—commercial travellers, theatrical artists, and the like—whose occupation necessarily demands migratory habits.

It is doubtful, however, whether any such modification will ever be entirely successful, for every departure from the panel system as originally designed involves a certain amount of personal care or forethought to the insured person in making application to his Insurance Committee for the appropriate forms and permission, and the doctors are occasioned extra clerical labour and account keeping. The trouble and labour is resented, and the result in many cases is that either the insured person goes without the medical treatment of which he is in need, or else pays for it out of his own pocket, when he is, strictly speaking, entitled to the attendance without payment.

### Approved Societies and the Panel System.

It is perhaps unwise to say too much on the subject of malingering at the present moment because an influential Committee has been appointed to investigate the matter, and the findings and recommendations of that Committee have not yet been published. At the same time it is asserted in friendly society circles by those whose past experience qualifies them to speak with authority that there is no possible doubt as to the fact of the sickness experience of the past few months having eclipsed all previous records. Friendly society leaders are talking gravely of the insolvency of their societies as an event in anticipation in a way they have never spoken before, and they appear to realise the necessity of immediate relief if the disappearance of the societies—particularly the smaller societies—as independent institutions apart from the National Insurance sections is to be averted. In the view of friendly society men, as expressed at the recent meetings of the National Conference of Friendly Societies, the seat of the mischief lies in the present administration of medical benefit. Prior to the advent of the National Insurance Act the societies managed their own medical benefits, appointing doctors to attend their members under contract practice. The National Insurance Act did away with this system, for the doctors resigned their appointments, and stipulated for the same terms for members outside the scope of the Act as they were granted for the State-insured members. It is not, however, so much on account of the extra charge now incurred that complaint is made by the societies, but rather because they no longer have the management of the benefit in their hands. The doctors' contracts are with the Insurance Committees, and although the majority of the members of such Committees are representatives of approved societies, there is not the same individual control by the societies themselves over the doctors as formerly.

By an unanimous vote the National Conference of Friendly Societies declared that the present position was unsatisfactory, and called upon the Government to allow the societies to revert to their former practice of making their own contracts for medical attendance and treatment of their members with full control of the doctors appointed. In view of the attitude of the medical profession towards contract practice and control by the societies when negotiating with the Chancellor of the Exchequer, there does not seem to be any likelihood of action being taken on this resolution of the Conference of Friendly Societies. It is clear, nevertheless, that the societies are not satisfied with the present panel system.

### The Dilemma of the London Insurance Committee.

This is not the most unsatisfactory feature of the present position. During the past few days it has become increasingly evident that the medical profession are not satisfied with their position under the Act. On the one hand we have to record the resignations of Wandsworth practitioners from the British Medical Association as a protest against the attitude of the Association in endeavouring to reconcile the interests of panel and non-panel doctors, a course which the retiring non-panel members consider to be impracticable. On the other hand, the expression of opinion by eminent medical men on the London Insurance Committee in connection with the dilemma in which that Committee is now placed regarding the distribution of the medical benefit fund clearly shows that, unless some way is speedily found whereby the fund can be distributed, there is likely to be trouble, and the panel doctors will relinquish their appointments.

It has from the outset been generally understood that the payment of the doctors for medical benefit, allowed on a capitation basis, was in the nature of an insurance premium. The doctor took a number of patients' names on his list, and held himself ready to attend them when ill, but was to be remunerated according to scale whether they were well or ill. Under the National Insurance Act, except for Ireland, medical benefit is provided for all insured persons. In consequence the Insurance Committees who are responsible for the administration of the benefit have been credited with the necessary funds, but either from apathy, or it may be antagonism to the Act, a substantial proportion of the insured persons have not even yet selected the doctor by whom they wish to be treated. Under the regulations of the Insurance Commissioners the choice should have been made by the end of the first quarter after medical benefit came into operation, so that ample time has been allowed in excess of the prescribed limit. The London Insurance Committee is particularly unfortunately situated. Out of a total of approximately 1,429,000 entered on the list of this Committee from the index slips supplied by the approved societies, it is estimated that about 400,000 have not yet chosen the panel doctors by whom they wish to be attended. The Act requires the adoption by every Insurance Committee of a system that will secure "the distribution amongst, and, so far as practicable, under arrangements made by, the several practitioners whose names are on the lists, of the insured persons who, after due notice have failed to make any selection, or who have been refused by the practitioner whom they have selected." With such a huge number of cases to deal with the Committee felt it beyond their power to make the distribution, and accordingly approached the Insurance Commissioners for instructions as to the disposal of the sum of £34,000 held on account of such cases for the first quarter.

### The Commissioners' Circular.

The Commissioners in a circular advised an impersonal distribution, neither the insured nor the doctor being aware of the name of the other. It was on the motion of Mr. P. Rockliffe that the legality of such a course was called in question, and when the Commissioners declined to submit the case to the law officers of the Crown the London Committee took the opinion of counsel (Mr. Danckwerts, K.C.), whose ruling is that the practitioners on the panel were entitled to be paid definite sums calculated at so much per head of the insured persons on their list, and they could not lawfully pay more, and were not entitled to make presents or distribute largess among the panel doctors.

This opinion would seem to indicate that some more satisfactory plan will have to be initiated for the distribution of the insured persons among the doctors on the panel in order that in future the whole of the fund may be apportioned. It would also preclude the disposal of the fund already in hand in respect of the past two quarters, and possibly also of the third quarter which ends on October 13. Until the matter is settled—and this may involve a decision of the Courts, or even an amending Act—the fund cannot be distributed.

London is an extreme example, but with very few exceptions all the other Insurance Committees are in the same dilemma, and it is estimated that by the end of the current year the total fund awaiting distribution will be about £1,000,000. This sum, in our opinion, undoubtedly belongs to the doctors as part of their remuneration, although they have not actually rendered any service to the particular insured persons involved.

How very different matters would have been if, instead of the capitation system, payment on the basis of attendances had been adopted! This system has been tried, notably in Manchester, and in the circumstances there seems every prospect of its extension to other Committee areas.

### A Possible Remedy.

Some remedy will have to be found in the long run for the present confusion. An important contribution to the discussion of the subject, though viewed from a somewhat different standpoint, appeared in our last week's issue in the section devoted to the National Medical Guild. The author, Dr. M. Milton Townsend, sets forth a scheme which would allow a cash alternative to medical benefit. Similar suggestions have been made previously, but in this case safeguards are inserted to ensure the application of the monetary equivalent towards medical treatment. It is possible that some such scheme will ultimately have to be adopted to give satisfaction to all parties concerned.

